# Pediatric Venous Thromboembolism in Orthopedic and Non-orthopedic Surgery

**DOI:** 10.7759/cureus.106434

**Published:** 2026-04-04

**Authors:** Aneesh V Samineni, Maria F Canizares, Danielle Cook, Kristin S Livingston, Colyn Watkins, Benjamin J Shore

**Affiliations:** 1 Orthopedic Surgery, Boston Children's Hospital, Boston, USA

**Keywords:** deep venous thrombosis, orthopaedic procedures, pediatric surgery, postoperative complications, pulmonary embolism, venous thromboembolism

## Abstract

Introduction

Venous thromboembolism (VTE) is a recognized postoperative complication in adults; however, limited data exist regarding VTE after surgery in children. The purpose of this study was to determine whether the incidence and associated clinical characteristics of pediatric VTE differ between orthopedic surgery-related (OSR) and non-orthopedic surgery-related (NSR) procedures.

Methods

Patients younger than 19 years with imaging-confirmed VTE (ultrasound (US) or computed tomography (CT)) within 30 days of an inpatient surgical procedure between January 1, 2009, and December 31, 2016, at Boston Children's Hospital, a single tertiary pediatric hospital in Boston, USA, were retrospectively identified. VTE was classified as OSR or NSR based on the index surgical procedure. The primary outcome was the incidence of VTE per 10,000 inpatient surgeries in the OSR and NSR cohorts. Secondary measures included VTE anatomic location and associated patient and procedural characteristics, including age, trauma, ambulatory status, central or peripherally inserted central catheter use, malignancy, infection, and clotting disorder or family history of VTE. Group differences were assessed using chi-square or Fisher’s exact tests with a significance level of 0.05.

Results

Among 233,339 inpatient surgical procedures, 86 VTE events were identified, corresponding to an overall incidence of 3.69 per 10,000 cases. The incidence was 5.56 per 10,000 in the OSR cohort and 3.22 per 10,000 in the NSR cohort. OSR VTEs occurred predominantly in the lower extremity, whereas NSR VTEs were more evenly distributed between the lower and upper extremities. Patients with OSR VTEs were older than those with NSR VTEs (mean age of 15.1 versus 7.9 years; p < 0.001). Trauma and non-ambulatory status were more common among OSR VTEs, whereas central or peripherally inserted central catheter use and malignancy were more frequent among NSR VTEs (all p < 0.05). Postoperative VTE prophylaxis was used more frequently in the NSR cohort than in the OSR cohort (p < 0.05).

Conclusion

Pediatric OSR and NSR VTEs differ in incidence, age distribution, anatomic pattern, and associated clinical context. Recognition of these cohort-specific profiles may support more informed perioperative assessment and vigilance in pediatric surgical patients.

## Introduction

Venous thromboembolism (VTE) in pediatric patients, although relatively rare, is concerning due to the associated increased risk of in-hospital morbidity and potential mortality. Furthermore, pediatric VTEs have been estimated to cost approximately $20,000 per event, totaling over $90 million each year [1]. There have been several studies investigating the incidence and risk factors associated with surgically related VTEs in adult patients [2-6]. However, the literature on VTE incidence in children is relatively small, with particularly limited work focused on surgically associated events. The overall incidence of VTE in pediatric surgery has been reported to be 20 per 10,000 cases, with the incidence of orthopaedic VTEs ranging from five to 10 per 10,000 cases and reported to be increasing over time [7-14].

Most studies reporting on surgical-related pediatric VTE arise from large databases, such as the Pediatric Hospital Information System (PHIS), the American College of Surgeons (ACS) National Surgical Quality Improvement Program-Pediatric (NSQIP-P) dataset, and the Kids’ Inpatient Database (KID). These databases primarily use International Classification of Diseases, 9th Revision (ICD-9) codes to classify cases [5,9,10]. Often, postoperative pediatric surgical VTE is coded in the secondary diagnosis position and missed when performing a primary diagnosis search. Secondary diagnosis codes have a positive predictive value (PPV) of only 75% for an acute venous thrombosis and as low as 50% for pulmonary embolism (PE) compared to a PPV of 95% when VTE is coded in the principal position [15]. As a result, relying exclusively on discharge diagnosis codes can significantly underestimate the true incidence of pediatric postoperative VTE.

While pediatric rates are much lower than adult VTE, there appears to be differential rates of VTE incidence across children admitted to the hospital for surgery. Samineni et al. [16] found that the incidence of pediatric orthopedic VTE (1.48 per 10,000) was significantly lower than that of non-orthopedic inpatients (5.55 per 10,000), highlighting significant differences in VTE risk factors and locations between surgical and non-surgical patients, but there has been little comparison among surgical cohorts [16]. Similarly, an analysis of the NSQIP-P database, which included 361,384 pediatric surgical patients across nine surgical disciplines, including orthopedic surgery, identified 378 cases of postoperative VTE, yielding an overall incidence of 10.5 per 10,000 patients. However, incidence rates varied significantly across surgical specialties, with neurosurgery having the highest incidence at 17.6 per 10,000, compared to 6.9 per 10,000 in orthopedic surgery [17].

These findings underscore the importance of recognizing that, while pediatric VTE is rare, certain surgical populations, particularly those undergoing non-orthopedic procedures, may have a higher incidence compared to their orthopedic counterparts. Accordingly, this study aimed to compare the incidence, anatomic distribution, associated risk factors, and prophylaxis patterns of postoperative VTE between orthopaedic surgery-related (OSR) and non-orthopaedic surgery-related (NSR) pediatric surgical procedures. We hypothesized that significant differences exist between OSR and NSR groups in terms of incidence, associated characteristics, and VTE location.

## Materials and methods

Patients under 19 years of age who were evaluated for VTE at Boston Children's Hospital, a tertiary pediatric hospital on Boston, USA, between January 1, 2009, and December 31, 2016, were identified through ultrasound (US) and computed tomography (CT) reports from the radiology department. VTE included ultrasound-confirmed deep venous thrombosis (DVT) and CT-confirmed PE; these were also analyzed descriptively as DVT versus PE when applicable. We then determined which patients had a diagnosis of VTE within 30 days after surgery. The most recent inpatient procedure was designated as the index surgery when multiple operations occurred.

Demographic, clinical, and prophylactic data were obtained from the medical records of these patients using a standardized electronic data collection form. Age at diagnosis was calculated in years using the date of the index VTE imaging study. VTE phenotype was classified as DVT only, PE only, or concurrent DVT and PE based on imaging-confirmed diagnoses, and the number of VTE events per patient within this window was counted. PE laterality (right, left, bilateral, or unknown) was abstracted from CT reports. DVT location was categorized as lower extremity, upper extremity, trunk, neck, or combination when more than one anatomic region was involved. For OSR cases, the index orthopaedic procedure was categorized from the operative note as trauma/fracture, trauma/ORIF, elective, or other. Prophylaxis was recorded from perioperative orders and the medication administration record and classified as mechanical (sequential compression devices) and/or pharmacologic anticoagulation, documented separately for pre- and postoperative use when available. Prophylaxis use reflected the treating service’s clinical judgment rather than a standardized institutional protocol.

Previously accepted published risk factors for VTE were recorded, including having a personal or family history of a clotting disorder [6,11], oral contraceptive (OCP) usage [18], infection [3,11], recent central or peripheral inserted central catheter (PICC) usage [11,13,14], obesity [13], trauma [14,16], cancer [2,3,19], and change in ambulatory status [8,16]. These factors were operationalized as follows: infection required a clinician-documented active infection and/or systemic antibiotics; clotting disorder/family history required documentation in the problem list, hematology notes, or admission history; change in ambulatory status required a new non-ambulatory status or restricted weight-bearing documented in therapy/nursing notes; and line exposure required a documented central/PICC line within 30 days. Obesity was defined as a body mass index at or above the 95th percentile. 

VTEs were further classified as orthopedic (OSR) if the VTE occurred within 30 days of undergoing orthopaedic surgery, or non-orthopedic (NSR) if the VTE occurred within 30 days of undergoing non-orthopaedic surgery. In other words, all patients included in this study underwent surgery within 30 days prior to the development of VTE. For incidence denominators, annual counts of inpatient surgical procedures were obtained from the hospital surgical database and classified as orthopaedic or non-orthopaedic using the performing department/service recorded for each operation.

The Boston Children's Hospital Institutional Review Board (IRB) reviewed and approved this retrospective analysis and authorized a waiver of informed consent under our institution’s ethical and regulatory framework (ref. no. IRB-P00025672).

Incidence rates for OSR and NSR VTE events were estimated for the eight-year study timeline along with 95% confidence intervals (CIs). OSR VTE incidence was calculated as the number of OSR VTE cases divided by the number of all orthopaedic surgical cases during the study timeline. A similar calculation was done for the NSR VTE incidence. A two-sample proportion test was used to determine if there was a significant difference in incidence rates between cohorts, with p < 0.05 considered significant. Patient demographics, VTE characteristics, and commonly reported risk factors were summarized and compared between the two surgical groups. Group differences were assessed using χ² tests, Fisher’s exact tests, independent-samples t-test, or Wilcoxon rank-sum tests, as appropriate. A yearly incidence of VTE (OSR, NSR, and total) was also calculated to determine if there were any trends in the data from 2009 to 2016. Missing data were handled using complete-case analysis; no imputation was performed, and denominators reflect available data. All statistical analyses were performed using SAS software (SAS Institute Inc., Cary, NC).

## Results

Eighty-six patients (57% female) with at least one surgically related VTE at an average age of 10.1 years (range, 0.0-18.9 years) were identified. A majority of patients were diagnosed with isolated DVT (79%; 68/86), followed by isolated PE diagnoses (13%; 11/86) and concomitant DVT/PE diagnoses (8%; 7/86). There were 26 OSR VTEs and 60 NSR VTEs. The frequency of DVT was significantly higher in the NSR cohort compared to the OSR cohort (85% vs. 65%, p = 0.04). Age at diagnosis differed significantly between the surgery groups (OSR = 15.1; NSR = 7.9 years; p < 0.001) (Table 1).

**Table 1:** Demographic characteristics of 86 patients included in the study Data are presented as mean ± SD (continuous) and n (%) (categorical). Wilcoxon rank-sum tests were used for non-normal continuous variables; Pearson’s χ² or Fisher’s exact tests were used for categorical variables, as appropriate. For Fisher’s exact tests the exact probability is computed, and no test statistic is reported. Sparse multi-level location variables are presented descriptively without inferential testing. Statistical significance was defined as p < 0.05. Z = Wilcoxon two-sample test, χ² = Pearson χ² tests, N/A: For Fisher's exact tests, no statistical test reported. SD: standard deviation, DVT: deep vein thrombosis, PE: pulmonary embolism, VTE: venous thromboembolism, ORIF: open reduction internal fixation.

	All subjects (n = 86)		Orthopedic (n = 26)		Non-orthopedic (n = 60)		Test statistic; p-value
Characteristic	Freq.	(%)	Freq.	(%)	Freq.	(%)	
Age at diagnosis (years; mean ± SD)	10.1	±6.80	15.1	±3.46	7.9	±6.73	Z = 4.47; *p < 0.001*
Sex (n, % female)	49	(57%)	14	(54%)	35	(58%)	χ²(1) = 0.15; p = 0.70
Only DVT (n, %)	68	(79%)	17	(65%)	51	(85%)	χ²(1) = 4.22;* p = 0.04*
Only PE (n, %)	11	(13%)	6	(23%)	5	(8%)	N/A; p = 0.08
DVT and PE (n, %)	7	(8%)	3	(12%)	4	(7%)	N/A; p = 0.43
Number of VTE's (n, %)							
1	71	(83%)	21	(81%)	50	(83%)	
2	12	(14%)	4	(15%)	8	(13%)	
3	3	(3%)	1	(4%)	2	(3%)	
PE location (n, %)	(n = 18)		(n = 9)		(n=9)		
Right	7	(39%)	6	(67%)	1	(11%)	
Left	5	(28%)	1	(11%)	4	(44%)	
Bilateral	5	(28%)	2	(22%)	3	(33%)	
Unknown	1	(6%)	0	(0%)	1	(11%)	
DVT location (n, %)	(n=75)		(n=20)		(n=55)		
Lower extremity	42	(56%)	18	(90%)	24	(44%)	
Upper extremity	26	(35%)	2	(10%)	24	(44%)	
Trunk	3	(4%)	0	(0%)	3	(5%)	
Neck	2	(3%)	0	(0%)	2	(4%)	
Combination	2	(3%)	0	(0%)	2	(4%)	
Orthopaedic event (n, %)							
Trauma/fracture			2	(8%)			
Trauma/ORIF			2	(8%)			
Elective			20	(77%)			
Other			2	(8%)			

During the study period, there were 86 patients with a VTE out of a total of 233,339 who underwent surgery (3.69 per 10,000 cases; 95% CI 2.97-4.57 per 10,000 cases). There were 26 OSR VTEs out of a total of 46,729 patients who underwent orthopaedic surgeries (5.56 per 10,000 cases; 95% CI 3.71-8.28 per 10,000 cases) and 60 cases with NSR VTEs out of a total of 186,610 patients who underwent non-orthopaedic surgeries (3.22 per 10,000 cases; 95% CI = 2.47-4.17 per 10,000 cases) (p = 0.03). Examining the OSR cohort, there were 20 patients with VTEs associated with elective orthopaedic surgery out of a total of 26,148 elective orthopaedic surgical procedures for an eight-year incidence rate of 7.65 per 10,000 cases, while six patients with VTEs associated with non-elective orthopaedic surgery out of a total of 20,581 non-elective orthopaedic surgical procedures yields an incidence rate of 2.92 per 10,000 cases (p = 0.05) (Table 2).

**Table 2:** Venous thromboembolism incidence rates for 2009-2016 Data are presented as counts (n), incidence rates per 10,000 procedures, and 95% confidence intervals (CIs). Between-group comparisons of incidence rates were performed using a two-sample test for equality of proportions with continuity correction (χ²). Statistical significance was defined as p < 0.05. *P-values correspond to comparisons between non-orthopedic surgery-related (NSR) versus orthopedic surgery-related (OSR) incidence and between elective orthopedic versus elective non-orthopedic procedures, respectively. DVT: deep vein thrombosis, PE: pulmonary embolism, CI: confidence interval

Category	DVT/PE patients	Total patients	Incidence rate (per 10,000)	Incidence (95% CI)	Test Statistic/ P-value*
Surgery-related	86	233,339	3.69	(2.97, 4.57)	
Non-ortho surgery-related	60	186,610	3.22	(2.47, 4.17)	χ²(1)=4.98; *p= 0.03*
Ortho surgery-related	26	46,729	5.56	(3.71, 8.28)	
Ortho (elective)	20	26,148	7.65	(4.80, 12.04)	χ²(1)=3.83.; p=0.05
Ortho (non-elective)	6	20,581	2.92	(1.19, 6.69)	

The distribution of DVT cases in the OSR group compared to the NSR group was significantly different, as most of the OSR DVT cases were in the lower extremity (90%), while 44% of the NSR DVT cases were in the lower extremity, and 44% of cases were in the upper extremity (p = 0.007). There were 21 DVT cases among the 20 OSR patients, of which 17 cases were due to elective surgery and four cases were due to non-elective surgery. Furthermore, 94% (16/17) of DVTs from elective surgery were located in the lower extremity, while 75% (3/4) of DVTs due to non-elective surgery were in the lower extremity. 

**Figure 1:**
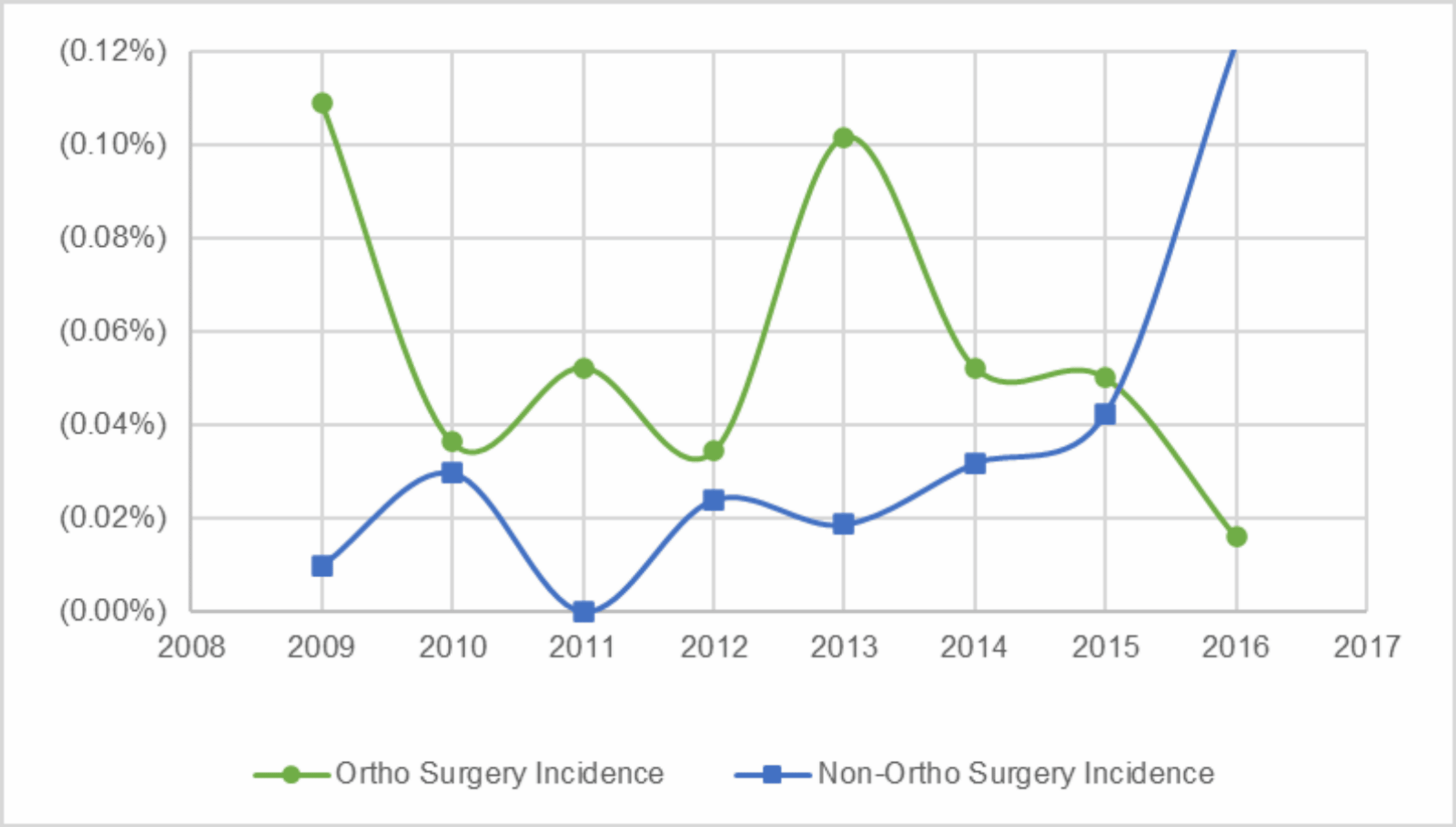
Annual incidence rates of orthopedic vs. non-orthopedic venous thromboembolism (VTE) surgeries

**Figure 2:**
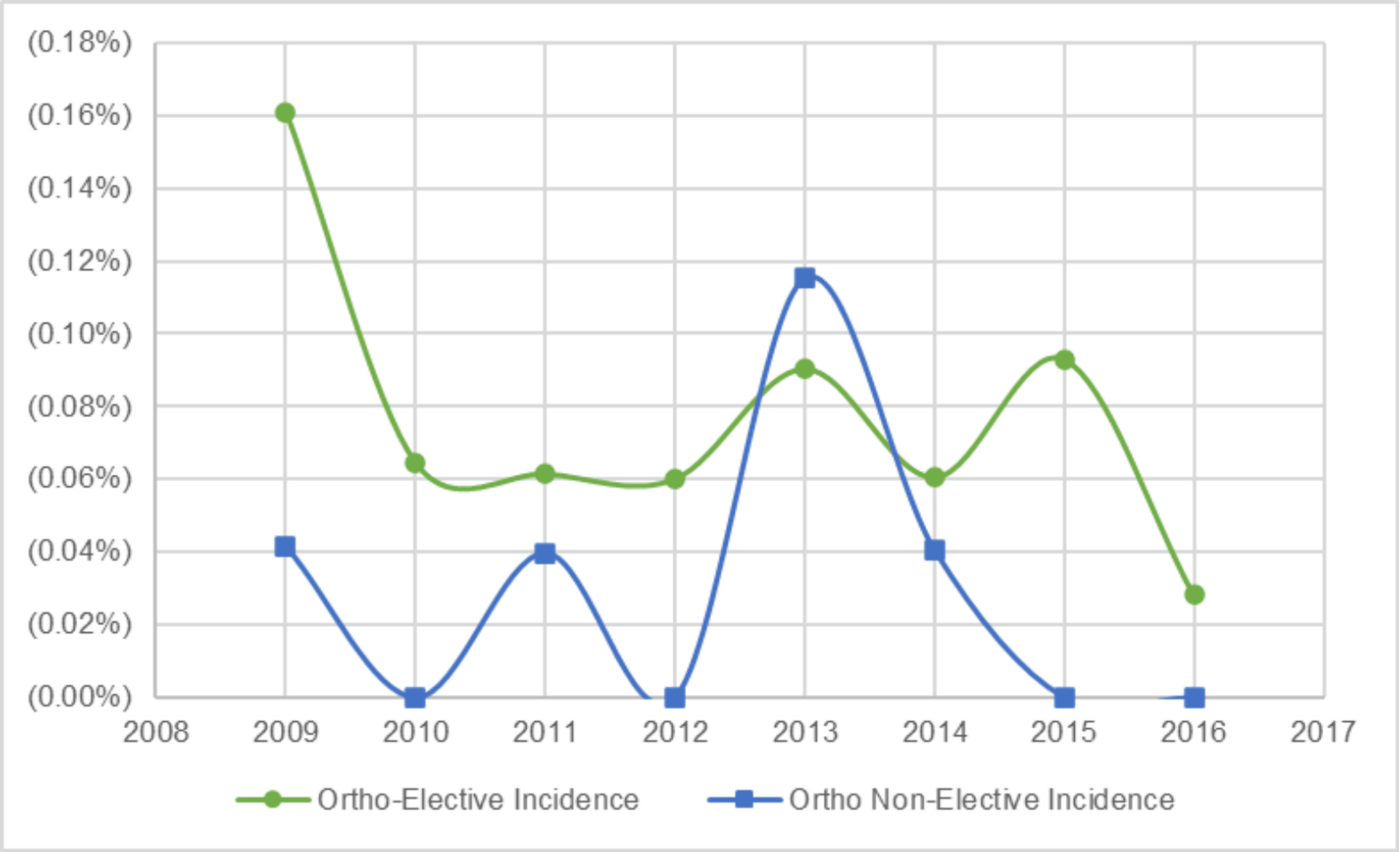
Annual incidence rates of elective vs. non-elective orthopaedic venous thromboembolism (VTE) surgeries

There were fluctuations in the annual VTE incidence for both the OSR and NSR groups. In 2009, the OSR VTE incidence was 10.9 per 10,000 cases, and the NSR VTE incidence was 0.9 per 10,000 cases. In 2016, the OSR VTE incidence decreased to 1.6 per 10,000 cases, and the NSR VTE incidence increased to 10.6 per 10,000 cases (Figure 1). The elective OSR VTE incidence rate fluctuated year to year from 2009, with 16 per 10,000 cases, to 2016 with three per 10,000 cases, while the non-elective OSR VTE incidence rate from 2009 to 2016 hovered around two per 10,000 cases (Figure 2).

**Figure 3:**
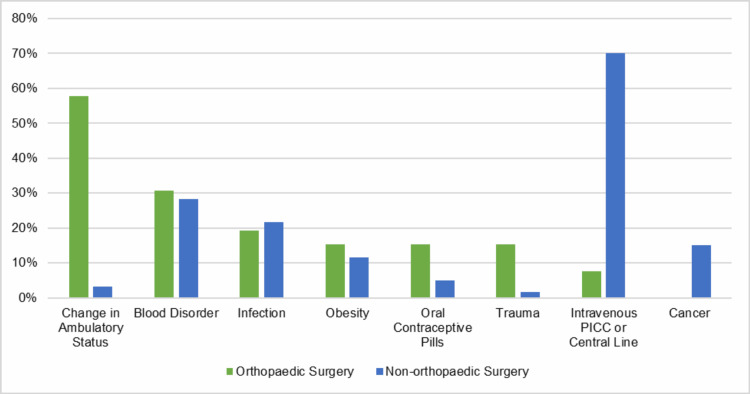
Percentage of demographic and clinical factors present in orthopaedic vs. non-orthopaedic venous thromboembolism (VTE) surgical patients

The clinical and procedural characteristics for OSR VTEs include non-ambulatory status (58%; p < 0.001) and trauma (15%; p = 0.03), while the main characteristics for NSR VTEs include the central/PICC line (70%; p < 0.001) (Table 3, Figure 3). Thirty-one percent of the OSR cohort also had a clotting disorder or positive family history of VTE, and 19% had an infection. Similarly, the NSR cohort had 28% of patients with a clotting disorder or positive family history of VTE, and 22% of patients had an infection.

**Table 3:** Demographic and clinical factors for surgical venous thromboembolism Data are presented as n (%). Pearson’s χ² or Fisher’s exact tests were used for categorical variables, as appropriate. For Fisher’s exact tests, the exact probability is computed, and no test statistic is reported. Sparse multi-level location variables are presented descriptively without inferential testing. Statistical significance was defined as p < 0.05. PICC: peripheral inserted central catheter, OCP: oral contraceptives. N/A: For Fisher's exact tests, no statistical test reported. χ² = Pearson chi-squared.

Characteristic	Orthopedic surgeries (n = 26)	Non-orthopedic surgeries (n = 60)	Test statistic; p-value
Infection	5 (19%)	13 (22%)	χ²(1) = 0.07; p = 0.8
Cancer	0 (0%)	9 (15%)	N/A; p = 0.05
Line	2 (8%)	42 (70%)	χ²(1) = 28.18; *p < 0.001*
Blood disorder	8 (31%)	17 (28%)	χ²(1) = 0.05; p = 0.82
Obesity	4 (15%)	7 (12%)	N/A; p = 0.73
OCPs	4 (15%)	3 (5%)	N/A; p = 0.19
Trauma	4 (15%)	1 (2%)	N/A; *p = 0.03*
Change in ambulatory status	15 (58%)	2 (3%)	χ²(1) = 33.80; *p < 0.001*

NSR VTE cases had a significantly higher rate of post-op (87% vs, 54%; p < 0.001) prophylaxis, compared to OSR (Table 4).

**Table 4:** Venous thromboembolism prophylaxis usage for those who had surgery Data are presented as n (%). Between-group comparisons were performed using Pearson’s χ² test when expected cell counts were adequate and Fisher’s exact test when expected counts were <5. For Fisher’s exact tests the exact probability is computed, and no test statistic is reported. Statistical significance was defined as p < 0.05. SCD: sequential compression device. N/A: For Fisher's exact tests, no statistical test reported.

	All surgeries (N = 86)		Orthopedic surgeries (N = 26)		Non-orthopedic surgeries (N = 60)		Test statistic/p-value
Characteristic	Freq.	(%)	Freq.	(%)	Freq.	(%)	
Pre-op prophylaxis	13	(15%)	2	(8%)	11	(18%)	N/A; p = 0.33
- SCD	4		1		3		
- Drug	8		1		7		
- SCD and drug	1		0		1		
Post-op prophylaxis	66	(77%)	14	(54%)	52	(87%)	χ²(1) = 10.95; *p < 0.001*
- SCD	5		1		4		
- Drug	57		13		44		
- SCD and drug	4		0		4		

## Discussion

Previous literature has established pediatric VTE as a rare event, and published rates align with the overall incidence rate of surgically-related VTEs (3.69 per 10,000 cases) [13,14]. The incidence of OSR VTEs was higher than that of NSR VTEs. Relevant clinical and procedural characteristics for OSR VTEs included decreased ambulatory status and trauma, while for NSR VTEs included central/ PICC line. While not significantly different between groups, the presence of a clotting disorder or family history of VTE was an important characteristic in both cohorts. The NSR cohort had significantly higher rates of post-op prophylaxis via anticoagulant medication and sequential compression devices, reflecting the differences in perioperative management between the two groups.

While VTE in pediatric patients has been less extensively studied compared to adults, the awareness of pediatric VTE has increased, as reflected by a recent systematic review of 30 studies on VTE in pediatric trauma or orthopedic patients that found a pooled incidence of 20 events per 10,000, with orthopedic-related VTE specifically at 16.6 events per 10,000 [13]. These rates are in line with a large NSQIP study that reports the incidence of pediatric VTE after surgery to be 20 cases per 10,000 surgeries and the incidence of pediatric VTE after specifically orthopaedic surgery to be 10 cases per 10,000 [7]. The present study reports a much lower surgery-related VTE incidence rate, 3.69 per 10,000 cases, compared to these reports. When it comes to trends across time, Sandoval et al. examined temporal patterns in pediatric DVT and reported an overall incidence of 9.7 events per 10,000 hospital admissions. During the study period (1995-2005), the VTE rate rose sharply from 0.3 to 28 per 10,000 admissions [11]. This study observed a similar fluctuation in VTE incidence over time, with OSR VTEs ranging from 10.9 per 10,000 cases in 2009 to 1.6 in 2016; and NSR VTEs ranging from 0.9 per 10,000 cases in 2009 to 10.6 in 2016. While the number of VTE cases per year was small, especially in the OSR cohort, these yearly incidence rates should be interpreted with caution, as small changes in the total number will have large changes in the observed rates, and the follow-up period is limited to only eight years, making it difficult to speculate if a true trend is occurring in this study

Increased patient age in the pediatric population has been cited to be associated with a higher risk of VTEs [17,20-23]. Sherrod et al. [7] showed that the risk of VTE was greatest in patients <3 years or >15 years. Georgopoulous et al. [10] reported that rates of VTE in elective orthopaedic surgery are stable until age 14 but have a sharp increase from ages 14-17. By separating patients in OSR and NSR cohorts, this study indicates the average age of patients with OSR VTEs to be 15.1 years, significantly higher than the average age of patients with NSR VTEs (7.9 years). This finding shows the importance of recognizing different characteristics present in the OSR and NSR cohorts that may not be seen in a cumulative analysis of all surgery-related VTEs.

By analyzing results from a follow-up survey, Sabharwal et al. [24] determined that the majority of pediatric DVTs occurred in the popliteal area or thigh and indicated that lower extremity surgery was one of the most commonly cited associations of VTE. Murphy et al. [8] expand on this, further specifying that the most common fracture locations of the lower extremity resulting in VTEs were femur or femoral neck (38.2%), and tibia/ankle (36.9%), followed by the pelvis (17.7%). This suggests that these surgeries were likely high-risk procedures. This aligns with the current study’s findings that the majority of DVTs in the OSR cohort (both elective and non-elective) occurred in the lower extremity.

Reported risk factors for surgically-related VTEs in pediatric patients have been determined from large database analyses and include ASA ≥3, longer operative times, and preoperative blood transfusion [7]. A recent study determined that risk factors for pediatric orthopaedic-related VTEs included having surgery, a decrease in ambulatory status, and trauma, while risk factors for non-orthopaedic-related VTEs included central/PICC lines; risk factors common to both cohorts included clotting disorder and family history of VTE [16]. In addition, a recent systematic review reported other risk factors as age, type and location of surgery, and obesity/BMI [13]. These findings indicate the primary clinical and procedural characteristics for OSR VTEs are older age, having trauma, and a decrease in ambulatory status. Primary characteristics for NSR VTEs are having a central line and malignancies, and factors for both cohorts include infection and blood disorder or family history of VTE. Notably, this study reported substantially higher rates of clotting disorder and family history than prior reports in pediatric VTE populations, where inherited thrombophilia and family history rates have been cited as 13% and 8%, respectively [25]. These differences in characteristics indicate the importance of having specific screening strategies tailored to the reason for the child’s hospital admission.

Sabharwal and Passannante’s 2011 survey results of POSNA members indicated that only 16% of respondents were familiar with VTE prophylactic institutional protocol; 77% of respondents reported utilizing mechanical prophylaxis, and 55% for chemical prophylaxis [26]. Murphy et al.’s 2020 survey results of POSNA members show that 33% of respondents were familiar with their institution’s VTE prophylaxis, and the most frequently cited risk factors for starting VTE prophylaxis were oral contraceptive usage, family history, and obesity. The newer survey study showed an increased percentage of respondents using mechanical prophylaxis (87%) and chemical prophylaxis (73%) [27]. Although there has been an increase in familiarity and treatment of pediatric VTE, the results of these surveys indicate the need for increased awareness.

Published guidelines for VTE prophylaxis indicate the use of mechanical prophylaxis for at-risk children, while pharmacological prophylaxis was contraindicated or used in high-risk children [28-30]. Although patients in the NSR cohort had significantly higher rates of postoperative prophylaxis, their recovery course was still complicated by VTEs after receiving higher rates of anticoagulation prophylaxis. Thus, this may indicate the highly thrombogenic nature of their conditions (cancer, presence of PICC/central line). Furthermore, the higher, though not statistically significant, incidence of VTE observed in OSR patients may reflect either a true increase in thrombotic risk associated with orthopaedic procedures or differences in baseline prophylaxis practices between OSR and NSR populations. Because this study was not designed to determine causality, these explanations cannot be confirmed. Nevertheless, among VTE cases in both cohorts, factors such as infection, a positive family history of VTE, and coagulopathy were observed, underscoring the importance of perioperative VTE risk assessment for all pediatric surgical candidates.

These findings should be interpreted in the context of several limitations. Most notably, the retrospective, single-center design is susceptible to selection bias that may limit external validity. The study period spans eight years, during which perioperative care pathways and prophylaxis practices evolved; these temporal changes may have influenced both the occurrence and detection of VTE. Because some older adolescents may have received care or imaging outside our system, a small number of events may not have been captured. Because postoperative VTE is so rare in children, some subgroup comparisons-especially within the orthopaedic cohort-are based on very small numbers and therefore sensitive to small changes in case counts. Variability in mechanical and pharmacologic prophylaxis use during the study period also limits the ability to directly attribute incidence differences to clinical or procedural factors alone.

Despite these limitations, this study offers several strengths that complement existing database-based research. Unlike administrative databases, which rely heavily on diagnosis codes with known limitations in accuracy, this cohort was derived from imaging-confirmed VTE events, with individual chart review of each case. This approach provides clinical granularity that large registries cannot offer, allowing more precise characterization of the contexts in which pediatric postoperative VTE occurs. Ultimately, while the sample size and single-center design limit broad generalizability, the detailed, imaging-verified dataset contributes meaningful clinical insight into a rare but serious complication. Larger, multicenter studies using standardized perioperative protocols will be essential to validate and build upon these findings.

## Conclusions

This study highlights clear differences in the age distribution, clinical characteristics, and anatomic patterns of VTE in orthopedic versus non-orthopedic surgical patients. While these findings cannot determine screening or prophylaxis strategies, they underscore the importance of recognizing the differing contexts in which VTE occurs. Further multicenter work is required to establish how these distinctions should guide clinical assessment and perioperative management.
